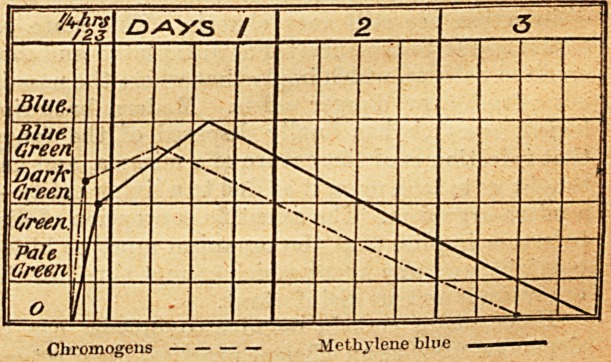# The Activity of the Renal Function

**Published:** 1907-01-05

**Authors:** J. W. Thomson Walker

**Affiliations:** Assistant Surgeon, North West London Hospital, and St. Peter's Hospital for Stone


### THE ACTIVITY OF THE RENAL FUNCTION.

### A Demonstration of the Methods Employed in its Estimation.

By J. W. THOMSON WALKER, M.B., C.M., F.R.C.S., Assistant Surgeon, North West London Hospital, and St. Peter's Hospital for Stone.

THIS demonstration was given at the Polyclinic, and indicated that the renal function should be tested in two types of cases. The first of these is where there is stricture, enlarged prostate, or a stone in a septic bladder; in such circumstances we want to determine the condition of the kidneys—whether they are able to withstand any severe operation, or, on the other hand, are so far damaged that the operation will probably turn the scale and cause death from suppression of urine.

The second type is that in which the patient has disease of one kidney, say a large pyonephrosis, and it is required to ascertain the condition of the other kidney. The question then arises whether the second kidney, that is, the one upon which the patient would have to depend, is sufficiently sound to carry on the entire renal function.

[Two kidneys were shown from a case of this nature. One kidney was represented by a large pyonephrosis, while the other kidney contained calculi. Methylene blue had been injected in this case, but was not eliminated by the affected kidney while the other organ passed it in a somewhat modified form. Nephrectomy was performed, but unfortunately the patient died from a septic condition ascending from the bladder to the remaining kidney.]

The method of testing the renal function in the first type of case is comparatively simple. All that is necessary is to give an injection of a known quantity of methylene blue, to note the time it takes for this to appear in the urine, and to estimate the total quantity eliminated. If, however, the action of one kidney has to be compared with that of the other, difficulties at once arise, because the function of each of the kidneys has to be tested separately by means of a catheter in each ureter. Some idea may be gained as to the defect of renal function on seeing the patient for the first time, although there may be very few symptoms of renal disease. One may observe dry tongue, thirst, headache, wasting, poorness of appetite, occasional vomiting, and with these, perhaps, a history of constipation. Such symptoms may be so slight as to cause little disturbance to the patient, and may be overlooked by the medical attendant. Again, the kidneys may be tender, especially in septic cases. If interference with the functions of the kidney is due to back-pressure only, the probability is that there will be no tenderness. As regards enlargement of the kidney, it is exceptional to find any enlargement from back pressure of urine—that is when the pressure is in front of the bladder. A person with enlarged prostate has very seldom an enlargement of the kidney, sufficient, at all events, to be felt through the abdominal wall.

The most reliable criterion for the activity of the renal function in surgical renal disease, apart from the tests here to be described, is the estimation of the amount of urea present in a twenty-four hours' sample of urine. The estimation should be repeated on several different days as it is unwise to trust to one estimation. The urea is constantly diminished in renal disease. The amount of diminution, however, is not a good criterion of the amount of the destruction of the kidney.

### SPECIAL TESTS.

There are three principal means of estimating the renal function artificially: (1) By the injection of methylene blue intramuscularly; (2) by the injection of phloridzin; (3) by cryoscopy.

1. In the methylene blue method 15 minims of a 5 per cent. solution of methylene blue are injected into the gluteal muscles. At the end of about fifteen minutes the urine passed will be found to be clear, but on being boiled with the addition of acetic acid it becomes blue, indicating that a certain body (chromogen) is present which can be converted in this way into methylene blue. Within half an hour the urine itself becomes blue and remains of that colour for between thirty to sixty hours, or even for several days.

The following table gives the different figures connected with the methylene blue method: (1) Elimination commences in thirty minutes; (2) chromogens appear in fifteen minutes; (3) quantity of methylene blue eliminated indicated by colour—dark green or blue; (4) duration from thirty-five to sixty hours; (5) the excretion may be constant or intermittent.

In a man with enlarged prostate from one to two hours may elapse before the methylene blue can be noticed in the urine. In order to test accurately the quantity of methylene blue eliminated a rather complicated colorimetric analysis is necessary. You can, however, form a very fair estimate of the excretion of blue by looking at the urine. The normal colour is dark green or Prussian blue, and the colour is at its height in the urine within five or six hours after the injection, continues at that for twenty-four hours, and then commences to fall. The following table indicates the curves of the chromogens and of the methylene blue:—

Chromogens – – – – Methylene blue ——

The chromogens should be estimated along with the blue otherwise the correct total quantity passed is not obtained. In some cases where the kidneys are diseased no methylene blue is passed, and only chromogens are eliminated. In other cases neither the one nor the other appears in the urine.

2. The Phloridzin test. This test is applied by injecting five milligrams of phloridzin under the skin of the patient. In fifteen minutes to half an hour the urine will be found to react to Fehling's solution as in diabetes. And this condition continues for about two to four hours. The striking point about this form of glycosuria is that there is more sugar excreted by the kidney than can be obtained from the amount of phloridzin employed. The sugar is formed from the blood by the renal cells and passed out. The phloridzin acts as a stimulant to the formation of sugar in the renal cells which act as gland cells in the same way as the cells of the mamma in forming sugar from the blood.

The following table gives the principal data connected with the phloridzin reaction: (1) Commences in thirty minutes; (2) duration two to four hours; (3) quantity of sugar eliminated 1 to 2 grammes; (4) limits of health lowest ½ to 1 gramme, highest 2-2.50 grammes.

The duration of the test is about two to four hours, and it has the great advantage over methylene blue that when the ureters are catheterised the catheter may be left in position until the reaction is completed. If the kidney is diseased less glycosuria is present, or there may be complete absence of elimination of sugar.

3. *Cryoscopy.*—The freezing point of a solution depends upon the density of the fluid, and this principle has been applied as a means of measuring the density of urine. In freezing the urine the freezing point varies according to its density. Normal urine freezes at 1.3° centigrade below the freezing point of distilled water. Where the patient has had a large draught of fluid it may rise to 1° centigrade below the freezing point of distilled water. When the patient's urine is concentrated it goes down as far as 2.30° centigrade.

The following table indicates the cryoscopy figures: (1) Urine 1.30° C. to 2.20° C.; (2) polyuria in health, 1.0° C.; (3) in concentrated urine, 2.30° C.

This method is merely another method of estimating the specific gravity, and it only differs from other methods in that it takes no account of the form of the molecules. In order to get the best results it is necessary to take the cryoscopy of the blood so as to get a balance between the freezing point of the blood and that of the urine. The most important observations are made by that comparison. The blood itself is constant in health. It is kept constant in density by the action of the kidneys, assisted by the sweat glands and the lungs, so that if the kidneys are acting badly a change will be got in the freezing point of the blood. When the kidneys are not acting the blood becomes denser and the freezing point gets lower. The constant freezing point of the blood is 0.56° C., and tables may be made out according to that. In many cases where other symptoms of renal disease are absent, and where the specific gravity and other tests fail, a comparison of the freezing points of the urine and the blood has indicated disease, and thus demonstrated the value of this method of observation.

## Figures and Tables

**Figure f1:**